# Flux Sampling Suggests Metabolic Signatures of High Antibody‐Producing CHO Cells

**DOI:** 10.1002/bit.28982

**Published:** 2025-04-11

**Authors:** Kate E. Meeson, Joanne Watson, Susan Rosser, Ellie Hawke, Andrew Pitt, Tessa Moses, Leon Pybus, Magnus Rattray, Alan J. Dickson, Jean‐Marc Schwartz

**Affiliations:** ^1^ Faculty of Biology, Medicine and Health University of Manchester Manchester UK; ^2^ Manchester Institute of Biotechnology University of Manchester Manchester UK; ^3^ EdinOmics, RRID:SCR_021838, Centre for Engineering Biology School of Biological Sciences, University of Edinburgh Edinburgh UK; ^4^ FUJIFILM Diosynth Biotechnologies Billingham UK

## Abstract

Chinese hamster ovary (CHO) cells remain the industry standard for producing numerous therapeutic proteins, particularly monoclonal antibodies (mAbs). However, achieving higher recombinant protein titers remains an ongoing challenge and a fundamental understanding of the cellular mechanism driving improved bioprocess performance remains elusive. To directly address these challenges and achieve substantial improvements, a more in‐depth understanding of cellular function within a bioprocess environment may be required. Over the past decade, significant advancements have been made in the building of genome‐scale metabolic models (GEMs) for CHO cells, bridging the gap between high information content 'omics data and the ability to perform *in silico* phenotypic predictions. Here, time‐course transcriptomics has been employed to constrain culture phase‐specific GEMs, representing the early exponential, late exponential, and stationary/death phases of CHO cell fed‐batch bioreactor culture. Temporal bioprocess data, including metabolite uptake and secretion rates, as well as growth and productivity, has been used to validate flux sampling results. Additionally, high mAb‐producing solutions have been identified and the metabolic signatures associated with improved mAb production have been hypothesized. Finally, constraint‐based modeling has been utilized to infer specific amino acids, cysteine, histidine, leucine, isoleucine, asparagine, and serine, which could drive increased mAb production and guide optimal media and feed formulations.

**Abbreviations**
FBA: flux balance analysis
FDBK: FUJIFILM Diosynth Biotechnologies, UK
FDBKA cell line: mAb expressing Apollo X CHO‐DG44 cell line
FDR: false discovery rate
FVA: flux variability analysis
GEM: genome‐scale model
GPR: gene–protein‐reaction rule
mAb: monoclonal antibody
Qp: specific productivity

## 1 Introduction

Unlike other rodent species, such as the house mouse (*Mus musculus*) or the Norway rat (*Rattus norvegicus*), the Chinese Hamster (*Cricetulus griseus*) is not primarily utilised for in vivo testing. Instead, its cells, CHO cells, are widely utilized as a secretory “cell factory” for the production of biopharmaceuticals. CHO cells are responsible for producing the majority of therapeutic mAbs (Xu et al. 2023), used to treat a range of illnesses, for example, cancer (Zahavi and Weiner 2020), autoimmune disorders such as Crohn's disease and rheumatoid arthritis (Hafeez et al. 2018), and potentially neurogenerative diseases such as Parkinson's disease (Castonguay et al. 2021; Pagano et al. 2024). The widespread use of CHO in the biopharmaceutical industry is attributable to several key factors. Their ability to grow in chemically defined suspension culture minimises the risk of contamination, reduces batch‐to‐batch variability, and facilitates large‐scale production. Additionally, CHO cells have a strong regulatory track record, are resistant to infection by human viruses and produce proteins with human‐like glycosylation patterns. Finally, CHO cells secrete proteins into the culture medium and optimised bioprocesses enable high yields of recombinant protein.

Traditionally, bioreactors for clinical mAb production have capacities of up to 250,000 L. Production typically occurs in fed‐batch mode over 14 days, with periodic nutrient additions. Some advanced fed‐batch processes have achieved titers exceeding 10 g/L (Xu et al. 2023). However, integrated and continuous bioprocesses (ICB) are gaining traction. This approach employs a continuous perfusion bioreactor coupled to a continuous downstream process, offering well‐documented advantages for both clinical and, in some cases, commercial manufacturing (Pybus et al. 2024).

Irrespective of the processing format, achieving high titers remains a persistent challenge in the quest for more cost‐effective biopharmaceuticals. Metabolic engineering through media and feed optimization has been a significant strategy to increase titers since the 1980s (Wurm 2004). However, the impact of these optimisations can vary widely. Factors such as the specific cell line (and even clones within a heterogenous cell population), the stage of bioreactor culture and the nature of the recombinant protein can influence the cellular response to a given media formulation.

While cell metabolism has been extensively manipulated to enhance CHO bioreactor productivity, the equilibrium of cellular metabolic demands has primarily been targeted through media blending and spent media analysis, coupled with one‐factor‐at‐a‐time (OFAT) and design of experiments (DoE)‐based approaches. These empirical methods demand extensive resources and lack insight into the complex and dynamic control of intracellular metabolism with the bioreactor environment. As metabolism supplies the “building blocks” for the protein product and the cellular factory required for its production, a deeper comprehension of CHO cell metabolism is crucial to identify metabolic bottlenecks and rationally guide more sophisticated cell and/or media engineering strategies (Gopalakrishnan et al. 2024; Kavoni et al. 2025; Park et al. 2024).

To address the evolving demands of biopharmaceutical manufacturing, the industry is embracing digital innovation to establish more streamlined, reliable, and cost‐effective bioprocess (Park et al. 2024). A deep mechanistic understanding of cellular behavior and dynamics is critical to creating realistic digital representations of bioprocesses. The sequencing of the CHO cell genome was therefore a pivotal moment, unlocking the genetic parts list which could be linked to cellular phenotypes (Xu et al. 2011). Additional 'omics analyses (e.g., transcriptomics, proteomics, and metabolomics) have allowed greater insight into cellular processes and phenotypes. These data enable the construction of GEMs, which mathematically bridge the gap between high‐information content 'omic data sets and phenotypes. Although maximising the variety and depth of 'omics datasets to form model constraints would result in a more personalised model, often transcriptomics datasets form the initial constraints, due to their coverage and availability. The size of transcriptomics datasets far exceeds other data types, offering tens of thousands of unique gene IDs compared to a few thousand proteins or up to a few hundred targeted metabolites, depending on the analytical platform. Thus, transcriptomics facilitates the tailoring of many more metabolic enzymes in the genome‐scale model. However, it is imperative that any model constrained with transcriptomics datasets be compared to experimental datasets informing cell behavior, to ensure the enzyme abundances have translated to phenotypic behavior in a reliable manner. CHO GEMs serve as digital twins, providing insight into cellular behavior and metabolic state within biomanufacturing processes (Park et al. 2021). This potentially enables systematic optimisation of bioprocesses and the identification of engineering targets *in silico*.

GEMs are computational models that can be constrained by various biological data, such as metabolite concentrations, flux measurements, and gene/protein expression levels, to narrow down the infinite solution space. Genome annotation and comprehensive literature review are crucial for constructing the stoichiometric matrix, which defines the reactants and products involved in metabolic reactions. Furthermore, gene–protein‐reaction (GPR) rules which describe the association among genes, proteins, and reactions are used to further constrain the model. Popular algorithms such as GIMME and iMAT can be employed to incorporate 'omic data sets (Becker and Palsson 2008; Zur et al. 2010).

Over the past decade, multiple GEMs have been developed for CHO cells, allowing constraint‐based workflows and metabolic predictions to guide bioprocess optimisation. In 2016, the foundational CHO GEM, iCHO1766, was introduced, accurately predicting CHO growth rates and CHO‐specific amino acid auxotrophies (Hefzi et al. 2016). This model was constructed by reconciling incomplete CHO GEMs and human metabolic models (Recon1 and Recon2 [Duarte et al. 2007; Thiele et al. 2013]). Building upon this foundation, three subsequent GEMs (iCHO2291, iCHO2048, and iCHO2101) were published (Fouladiha et al. 2021; Gutierrez et al. 2020; Yeo et al. 2020). The most recent and comprehensive CHO GEM, iCHO2441, incorporates updated elements and has been systematically evaluated against all the previous models (Strain et al. 2023). These GEMs serve as platforms for constraint‐based modeling, allowing researchers to tailor models to specific experimental conditions and generate personalised metabolic predictions.

GEMs have many applications in bioprocess optimization, including improved media design through *in silico* simulation of nutrient additions and supplementation and the identification of targets for cell engineering based on predicted phenotypes. For example, constraint‐based modeling has been used to propose optimal media conditions that extend the cell growth phase (Ramos et al. 2022), to calculate the energetic costs of protein secretion (Gutierrez et al. 2020) and to predict amino acid concentrations in culture using machine learning (Schinn et al. 2021). However, despite their great potential for the rational design of CHO cell lines and associated cell culture processes, GEMs continue to face challenges and limitations in terms of model reliability, method development, and practical application.

Flux balance analysis (FBA) is a common method to predict metabolic fluxes within a GEM, identifying a single optimal steady‐state solution based on an “objective function” (Orth et al. 2010). However, to explore a range of possible solutions, flux variability analysis (FVA) can be employed, determining the maximal and minimum flux values for reactions while satisfying given constraints (Mahadevan and Schilling 2003). The most common objective function for FBA and FVA is cell growth, especially in cancer constraint‐based modeling (Yizhak et al. 2015). However, in the case of CHO cell modeling, the choice of an appropriate objective function becomes more complicated. Given the distinct growth and productivity phases of CHO cell culture, it is ambiguous at any given stage whether the cell is focussed on either solely growth or protein production, or a combination of both processes. Therefore, to avoid the oversimplification and errors that optimising an inappropriate objective function could represent, we adopt an alternative flux analysis method that does not require an objective function. In the flux sampling framework, the model is solved in an unbiased manner with fluxes sampled from an ensemble of steady‐state solutions. The marginal flux distribution can be summarised for each reaction as a histogram and can also be investigated for flux correlations (Herrmann et al. 2019). The coordinate hit‐and‐run with rounding algorithm has been determined to be the most efficient flux sampling algorithm (Haraldsdóttir et al. 2017; Herrmann et al. 2019), and previously, flux sampling has been used to evaluate the accuracy of the CHO GEM, iCHO2441 (Strain et al. 2023) and to predict uptake and secretion behavior across distinct culture phases (Gopalakrishnan et al. 2024). However, due to the computational intensiveness of flux sampling, it is used much less often than FBA and FVA, highlighting the need for new workflows employing the methodology. Furthermore, due to its unbiased nature, flux sampling represents the ideal analysis method for studying the metabolic plasticity of CHO cells.

Utilising the iCHO2441 model as a framework (Strain et al. 2023), we aimed to constrain this CHO GEM with transcriptomics data to generate three separate culture phase‐specific models. By comparing these models, we sought to identify metabolic features associated with improved mAb production and identify potential targets for directed media and feed optimisation. Our hypothesis was that flux sampling of constraint‐based models could highlight metabolic features associated with high mAb production, and that these flux predictions would be representative of experimental bioprocess data. The practical objective here was to develop a flux sampling workflow which could identify high mAb producing solutions and to use statistical methods to uncover significant metabolic signatures of high mAb producing cells and characterise distinct phases of CHO cell bioreactor culture.

## 2 Materials and Methods

### 2.1 Experimental Materials and Methods

#### 2.1.1 Cell Line Details and Growth Conditions

The cell line used for experimental work was a mAb expressing Apollo X CHO‐DG44 cell line (FUJIFILM Diosynth Biotechnologies), which will be referred to as “FDBKA”. Fed‐batch cultures of FDBKA were performed in a 2 L BIOSTAT single‐use bioreactor (Sartorius) with an initial working volume of 1.2 L JM05B media (FUJIFILM Irvine Scientific, catalogue number 981486) at a starting density of 0.5 × 10^6^ cells/mL. All the cell cultures were performed in triplicate. The pH of culture was maintained at 7.0 ± 0.05 using carbon‐dioxide gas injection and sodium carbonate base addition. Temperature was set at 37°C and controlled using a bioreactor jacket. Dissolved oxygen was maintained at 40% of air saturation. The feeding regime consisted of daily additions of l‐glutamine (Merck, catalogue number 1002861000), HyClone Cell Boost 7a Supplement (Cytiva, catalogue number SH31026.08), and HyClone Cell Boost 7b Supplement (Cytiva, catalogue number SH31027.09). Glucose concentrations were maintained above a threshold value using a 50% (w/v) concentrated solution (SAFC, catalogue number CR40138‐20B). l‐glutamine was also maintained above a threshold value using a 200 mM solution.

#### 2.1.2 Bioprocess Measurements

For metabolite analysis of the cell culture, daily measurements were taken informing lactate and ammonia production (Bioprofile Flex 2; Nova Biomedical). Growth rate was interpreted from viable cell count (ViCell XR; Beckman Coulter), and product concentration was measured using a Protein A UPLC‐based method.

#### 2.1.3 Transcriptomics Generation and Analysis

Samples were taken for the transcriptomics data set between days 4 and 14 of cell culture (days 4, 5, 6, 7, 8, 11, 12, and 14); there were two technical repeats per sample across three separate bioreactors, (representing biological repeats). FDBKA cells were grown in JM05B media conditions, provided with high glutamine concentrations. The samples went through automated preparation using TruSeq stranded mRNA‐seq library and sequenced using NovaSeq. 100PE. Reads were trimmed, and aligned to the *C. griseus* genome (222‐107), which was assembled at the Edinburgh Genomics Facility, using STAR version 2.7.3a (Dobin et al. 2013) specifying paired‐end reads and the option—outSAMtype BAM Unsorted (all the other parameters were left at default). The quasi‐alignment‐based tool Salmon was used for transcriptome quantification (Patro et al. 2017), and the raw counts table was filtered to remove genes consisting of near‐zero counts, filtering on counts per million. The transcript level abundances were summed into gene level abundances using the bioconductor package tximport (Soneson et al. 2015). Reads were normalised using the weighted trimmed mean of M‐values method (Robinson and Oshlack 2010).

The distribution of the normalized transcriptomics data set was visualised using principal components analysis (PCA). DESeq2 differential gene expression analysis was performed (in R) between transcriptomics samples from different points in the time series (Love et al. 2014), including between concurrent time points, for example, day 4 to day 6, or between distinct culture phases, for example, day 4 to day 12 (Table 1). Differential expression was defined as an adjusted *p*‐value (false discovery rate; FDR) of less than 0.05 and a minimum fold change of 2. Heatmap visualisation (using *Z*‐scores) was applied to subset of differentially expressed genes, which were selected according to differential expression between concurrent time points, regardless of direction, that is, day 4 to day 6, as these were two neighbouring measurements, but this subset did not include those genes differentially expressed between day 4 and day 12. Genes included in the heatmap were carried forward to functional enrichment analysis via the biomaRt R package, specifying the “KEGG_2021_Mouse” reference genome and the “GO_Biological_Process_2023” ontology (Durinck et al. 2005, 2009).

**Table 1:** Contrasts used for DESeq. 2 analysis. Each differential expression analysis is between two individual days, as specified by Contrast A and Contrast B.

Variable	Contrast A	Contrast B
Day	Day 4	Day 6
Day	Day 4	Day 8
Day	Day 4	Day 12
Day	Day 6	Day 7
Day	Day 7	Day 8
Day	Day 8	Day 11
Day	Day 11	Day 12
Day	Day 12	Day 14

### 2.2 Computational Materials and Methods

Code for constraint‐based analysis methods detailed in this project is available at the corresponding GitHub: https://github.com/katemeeson/CHO_cell_modelling_2024/tree/main. A combination of R and Python were used across this project.

#### 2.2.1 Adapting the iCHO2441 Genome‐Scale Model

The iCHO2441 GEM (Strain et al. 2023) was downloaded from BioModels and the reactions relating to mAb production were updated to represent the product profile of the FDBKA cell line. Peptide sequences for heavy and light chains were converted into the reaction definition and used to estimate ATP and GTP hydrolysis. In the adapted version of iCHO2441 used here, the reaction entitled “ICproduct_TRANSLATION_protein” relates to product formation. The updated iCHO2441 was checked for consistency and errors using the “validate_sbml_model” function in COBRA (Ebrahim et al. 2013), and *in silico* media conditions were left at their default values.

During the curation of iCHO2441 (Strain et al. 2023), metadata describing metabolic subsystems and compartments was taken from iCHOv1 (Hefzi et al. 2016) and mapped to the new GEM; this has facilitated a subsystem enrichment analysis, which has been demonstrated in Section Results.

#### 2.2.2 Integrating Transcriptomics Into GEM

Before integration as model constraints, the transcriptomics gene IDs had to be mapped to the model IDs. The iCHO2441 GEM uses NCBI gene IDs, and these were mapped to Ensembl Gene IDs using a combination of the BioMart web‐based tool (Durinck et al. 2005, 2009) and flat files from NCBI, Ensembl, and Uniprot. The iCHO2441 GEM also uses slightly altered BIGG IDs for metabolites, therefore these have been adapted using mappings against KEGG, CHEBI, and other flat files from the BIGG website (Hastings et al. 2016; Kanehisa et al. 2022; Kanehisa 2000) (http://bigg.ucsd.edu/).

Before transcriptomics was integrated into the GEM, visualisation indicated that day 5 could be a batch effect, therefore it was removed from further analysis (Figure S1). Gene expression values were averaged across the days to be grouped to form the three distinct time phase models (early exponential: day 4; late exponential: day 6, 7, and 8; stationary/death: day 11, 12, and 14) and these were translated to reaction constraints using a previously published algorithm (Timouma et al. 2024). This 'omics integration algorithm enforces gene expression values as constraints for the lower and upper bounds of reactions within a GEM, and integration is directed by reaction reversibility and the GPRs in the GEM (Timouma et al. 2024). Reactions with genes not included in the transcriptomics were left unconstrained—an approach employed by other, existing algorithms, such as PROM (Chandrasekaran and Price 2010)—because the removal of all these reactions would make the model infeasible to solve.

#### 2.2.3 Flux Sampling and Isolating the High mAb‐Producing Solutions

For all the methods, the Gurobi optimiser was used with an academic license (Gurobi Optimization LLC. 2023). Using the cobra *sample* function (Ebrahim et al. 2013), each of the time phase models were sampled 50,000,000 times and solutions were stored every 10,000 iterations, resulting in 5000 data points per reaction, per model. This was the same approach taken by Strain et al. (2023), and helps prevent oversampling of the same area of solution space (Herrmann et al. 2019). The coordinate hit‐and‐run with rounding algorithm was used for flux sampling (Haraldsdóttir et al. 2017).

To identify reactions associated with high mAb‐production, solutions were selected based on a 95th percentile threshold of mAb production; this meant the metabolic profile of 250 high mAb‐producing solutions were compared against a pool of 5000 solutions, per culture phase‐specific model. The Mann–Whitney *U* test was implemented using the *mannwhitneyu* function from the *scipy.stats* library (Virtanen et al. 2020). The null hypothesis was that the flux distribution would not change between the high mAb‐producing solutions and all other solutions. *p*‐values were adjusted using the *fdrcorrection* function from the *scipy.stats* library (Virtanen et al. 2020). Reactions with a false discover rate (FDR) of less than 0.05 were considered to have skewed flux in the high mAb‐producing solutions.

## 3 Results

**Figure 1:**
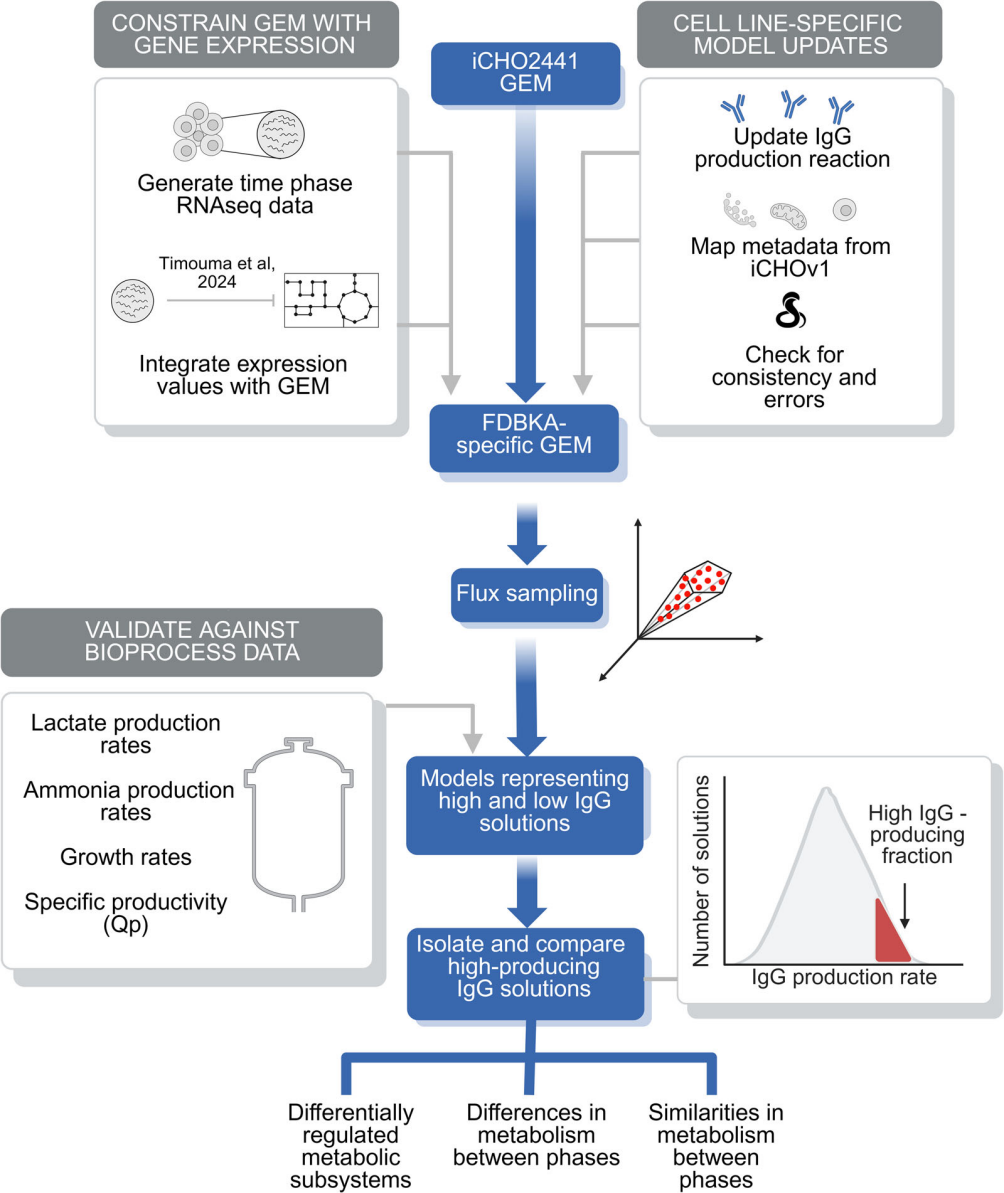
Workflow for obtaining biological conclusions from flux sampling results. Time series transcriptomics has been integrated into an updated version of iCHO2441 (Strain et al. 2023), using a recently designed universal integration algorithm (Timouma et al. 2024). Flux sampling has been applied to three culture phase‐specific constraint‐based models and metabolic predictions have been validated against bioprocess data from multiple bioreactors. High mAb‐producing solutions have been extracted from *n* = 5000 flux sampling solutions, and reactions exhibiting a change of flux which was statistically significantly associated with high production have been used to predict metabolic signatures. Qp: cell‐specific productivity.

For this project, the iCHO2441 model (Strain et al. 2023) was adapted to represent the specific mAb produced by an industrially relevant FDBKA CHO cell line, providing a scaffold for transcriptomic data integration (Figure 1). The mAb translation reaction and product composition (“ICproduct_TRANSLATION_protein”) required all the 20 amino acids, ATP, GTP, and H_2_O to produce the desired protein product, and produced, ADP, AMP, GDP, protons, inorganic phosphate, and pyrophosphate. Separately, the reaction corresponding to production of the mature mAb product (ID “ICproduct_Final_demand”) was primarily used to study protein production. Following model adaptation, flux sampling was performed on three transcriptomics‐constrained models, each representing a distinct culture phase of CHO cells (Figure 1). These models were validated using bioprocess data and used to predict the metabolic features of high mAb‐producing CHO cells (Figure 1).

### 3.1 Transcriptomics Analysis Predicts Features of Distinct CHO Cell Culture Phases

Before its integration into the adapted iCHO2441 model, the quality and depth of the transcriptomics data was analysed. After mapping to the genome and trimming, there were 20.6–34.4 M read pairs which remained, representing 53.3–61.4% of the initial total read pairs—with a total of 22,220 unique genes detected after filtering.

**Figure 2:**
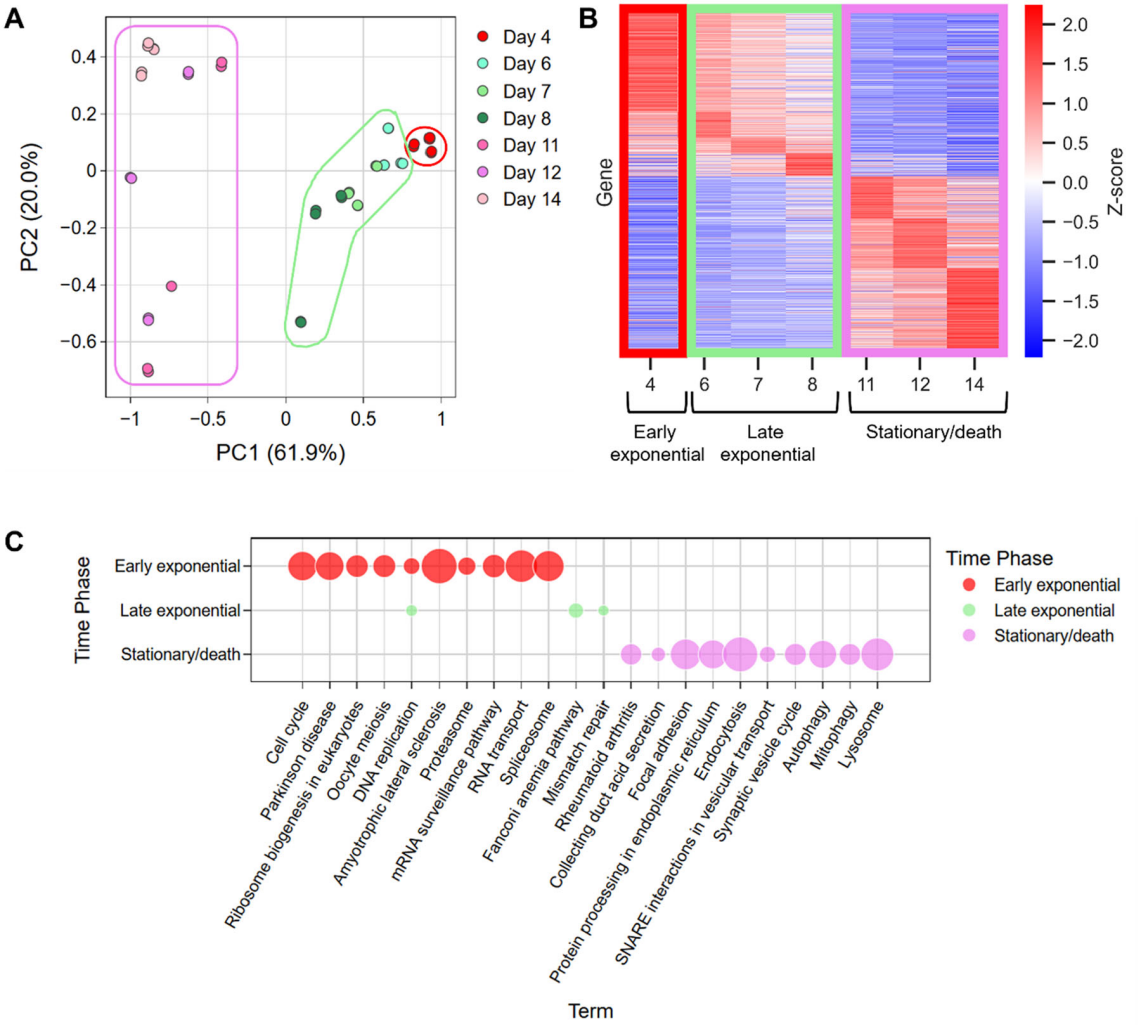
Gene expression represents distinct culture phases. A. PCA of time series transcriptomics. Individual data points for the same day represent two technical replicates across three biological replicates (separate bioreactors) B. Heatmap for the expression of genes found to be differentially expressed at concurrent time points, regardless of direction, according to DESeq. 2 analysis (FDR < 0.05 and a minimum fold‐change of 2). Days have been grouped into early exponential (red), late exponential (green) and stationary/death phase (purple), according to gene expression. Z‐score shown on heatmap. Genes ordered according to time phase in which gene expression peaked. C. Functional enrichment of differentially expressed genes from B, with GO Biological Process annotated on *x*‐axis. Size of bubble corresponds to number of differentially expressed genes from this GO term. Notebooks: DeSeq. 2 analysis, and enrichment analysis on DeSeq. 2_analysis.R; PCA performed, heatmap generated, and enrichment results visualized on transcriptomics_figs.ipynb.

The time series transcriptomics samples demonstrated a linear trend over time, with the most variation having been observed between samples taken at day 4 and stationary/death phase time points (days 11, 12, and 14) (Figure 2A). Owing to the divergence in gene expression observed over time, the most differentially expressed genes were identified between days 4 and 12, where there were 2417 genes upregulated and 1306 downregulated at day 12 compared to day 4, respectively (Figure 2B)—representing approximately 16.8% of all unique genes detected. Concerning the difference in gene expression between early exponential and late exponential, there were 612 genes upregulated and 129 downregulated at day 8 compared to day 4 (Figure 2B). Furthermore, as the CHO cells moved into the stationary/death phase, there were 623 genes upregulated and 457 downregulated at day 11 compared to day 8 (Figure 2B). In summary, differential gene analysis indicated that the greatest change in gene expression occurred at the transition from the early exponential to the late exponential phase, but there were differentially expressed genes at all time points.

The next task was to interpret the differential gene expression between CHO cell culture phases, to show that changes in gene expression translated to a meaningful culture phenotype, therefore justifying the integration of transcriptomics into the iCHO2441 GEM for metabolic analysis. Genes showing a peak expression at day 4 were enriched for cell cycle, ribosomal, and nucleic acid processes (Figure 2C), which could be interpreted as cell growth. Similarly, genes showing a peak expression at days 6, 7, and 8 were enriched with DNA replication and mismatch repairs processes (Figure 2C). In contrast, genes with peak expression at the stationary/death phase were enriched for cell death and cellular stress pathways, including autophagy, mitophagy, and lysosome pathways (Figure 2C).

### 3.2 Comparing Culture Phase‐Specific Model Predictions With Bioprocess Data

**Figure 3:**
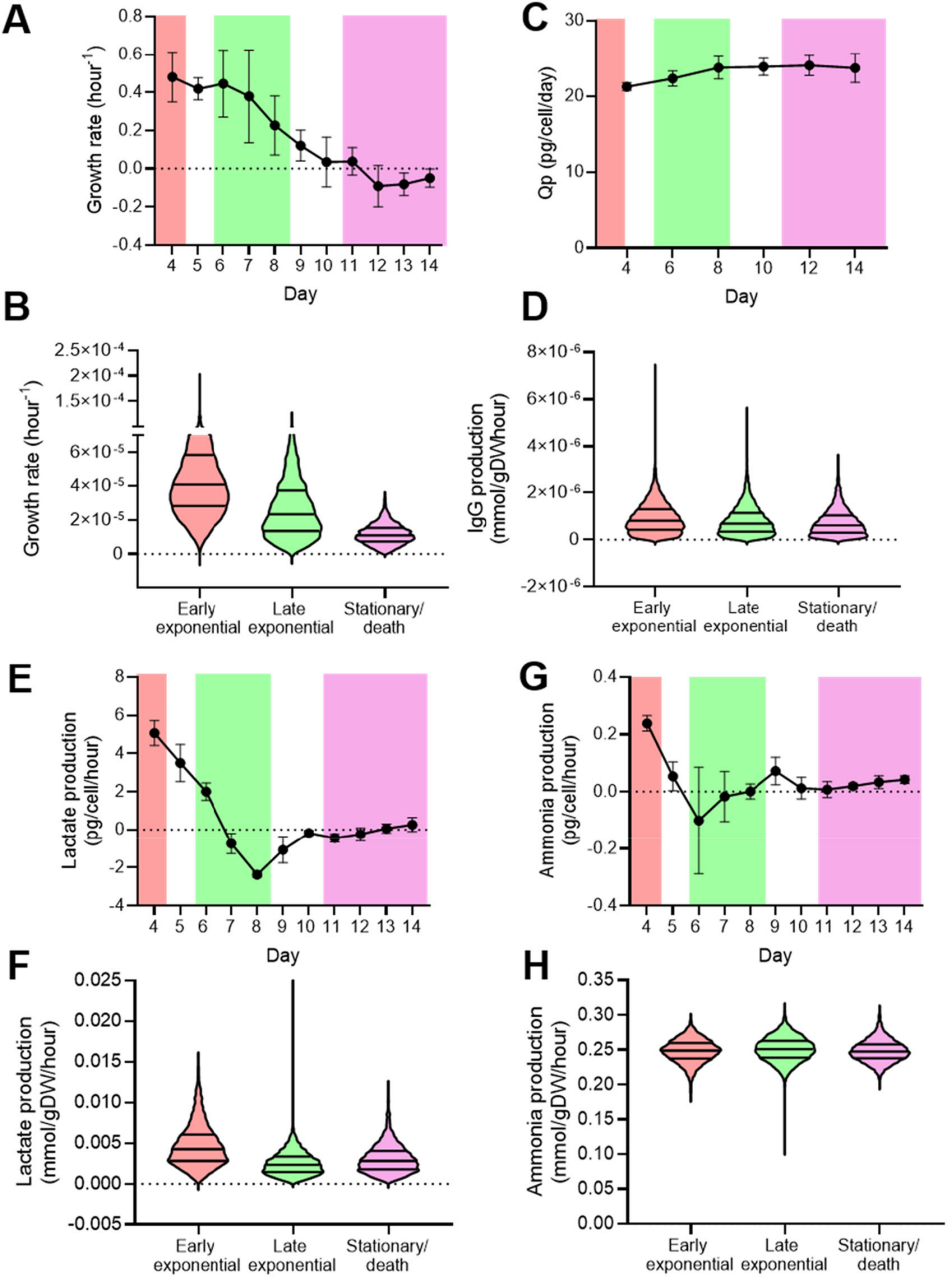
Validating metabolic predictions using bioprocess data from industrial bioreactors. It is possible to convert between pg/cell/h (bioprocess units) and mmol/gDW/h (constraint‐based model units) using reported dry cell weights, which vary per CHO cell line and condition (Széliová et al. 2020), and the molecular weight of the metabolite. The following formula should be used for this conversion: $\left(\frac{\alpha *10^{-12}}{\beta }\right)*(1000/\gamma )$, where α is rate of bioreactor process (pg/cell/h), β is dry cell weight (g) and γ is M_r_ of metabolite (g/mol). A. Growth rate measured from bioreactors at FDBK (hour^−1^). Error bars correspond to one standard deviation from the mean of six biological replicates (individual bioreactors). Culture phase as determined by gene expression (either early exponential, late exponential or stationary/death) has been overlayed onto the graphs for ease of comparison to model predictions. Negative values indicate cell number is decreasing over time, i.e. through cell death. B. Constraint‐based modeling predictions of cellular growth rate (hour^−1^). C. Specific productivity (Qp) measurements from bioreactors (pg/cell/day). Six biological replicates. D. Constraint‐based modeling predictions of the mAb production rate (mmol/gDW/h). E. Rate of lactate production (pg/cell/h). Positive values correspond to metabolite production and negative to consumption. F. Constraint‐based modeling predictions of the rate of lactate production (mmol/gDW/h), taken from model.summary() in COBRApy. Median, 25th and 75th percentile have been indicated. G. Rate of ammonia production as measure from bioreactors (pg/cell/h). Six biological replicates. H. Constraint‐based modeling predictions of the rate of ammonia production (mmol/gDW/h).

After integration into the adapted iCHO2441 model, it was necessary to experimentally validate the key metabolic predictions of the three culture phase‐specific models (Figure 3). Bioprocess measurements included the following: growth, specific productivity (Qp), and lactate and ammonia production. Bioprocess measurements were compared to model predictions, following flux sampling (Figure 3).

It was important for modeling to accurately predict CHO cell growth rate, which experimentally, was shown to decrease over time from the early and late exponential phases to negative growth rates as cells move into the stationary/death phase (Figure 3A). Constraint‐based modeling was able to recapitulate this behavior, where the median growth rate decreased by approximately 40% between early and late exponential, and by 50% between the late exponential and stationary/death phases (Figure 3B).

Models should accurately represent the mAb production behavior of CHO cells. Compared to growth rate, which experimentally showed a decrease over time, the specific productivity was found to be constant, as evidenced by overlapping standard deviation (Figure 3C). Typically, CHO cells show an increased productivity with decreased growth rate as they shift through the culture phases, and cell cycle arrest at the later culture phases is one approach to enhancing specific productivity of CHO cells (Kumar et al. 2007). However, an increase in specific productivity at the late exponential and stationary/death phases was not observed in the FDBKA cell line, indicating a cell line‐specific phenotype (Figure 3C). Modeling predicted a consistent productivity over time, where although there was a decrease in the upper ranges of flux sampling solutions across the culture phase models (Figure 3D), the standard deviation of mAb production overlapped between all culture phase models.

CHO DG44 cells switch from lactate production to consumption over time regardless of media composition (Reinhart et al. 2015), and this has been represented by the bioprocess data, where a negative reaction flux indicated consumption from day 7 onwards (Figure 3e). When constrained with transcriptomics data, the culture phase‐specific GEMs were able to capture lactate production at the early exponential phase, and although not able to predict consumption at the later culture phases, they did predict a shift towards negative values between the early and late exponential culture phases, represented by the quartiles of the flux sampling solutions (Figure 3f). Furthermore, the models also captured the increase to more positive values, which was observed experimentally between the late exponential and stationary/death culture phases (Figure 3f). The fact that models were not able to capture the lactate consumption phenotype is suspected to be because the late exponential phase (days 6, 7, and 8) represents a transition state for the cells, where at day 6 cells are producing lactate and day 8 consuming lactate (Figure 3e), which is hard to capture *in silico*, where gene expression data used to constrain the model was averaged across the 3 days (Figure 3f).

Experimentally, the pattern of ammonia production over time follows a similar trend to lactate production, where there is a decrease in production over time (Figure 3g). Ammonia production in CHO DG44 is mainly due to the deamination of glutamine consumed from the feed source, and glutaminolysis occurs at an increased rate at the early exponential phase (Ahn and Antoniewicz 2011; Coulet et al. 2022). Although the models have not been sensitive to this decrease in ammonia production over time, they have been able to capture the increased variability in ammonia production behavior at the late exponential time phase, which is evidenced by a large standard deviation at day 6 (Figure 3g) and range of flux sampling solutions which was the greatest for the late exponential model (0.2 mmol/gDW/h compared to 0.11) (Figure 3h).

### 3.3 Differential Regulation of Metabolic Subsystems at Each Culture Phase

To begin with, the relationship between growth (reaction ID: “biomass_cho_prod”) and productivity (reaction ID: “ICproduct_Final_demand”) was explored across 5000 flux sampling solutions. In agreement with the bioprocess data there was no observable change in mAb production rate solutions as the corresponding growth rate solution increased, per culture phase (Figure S2).

**Figure 4:**
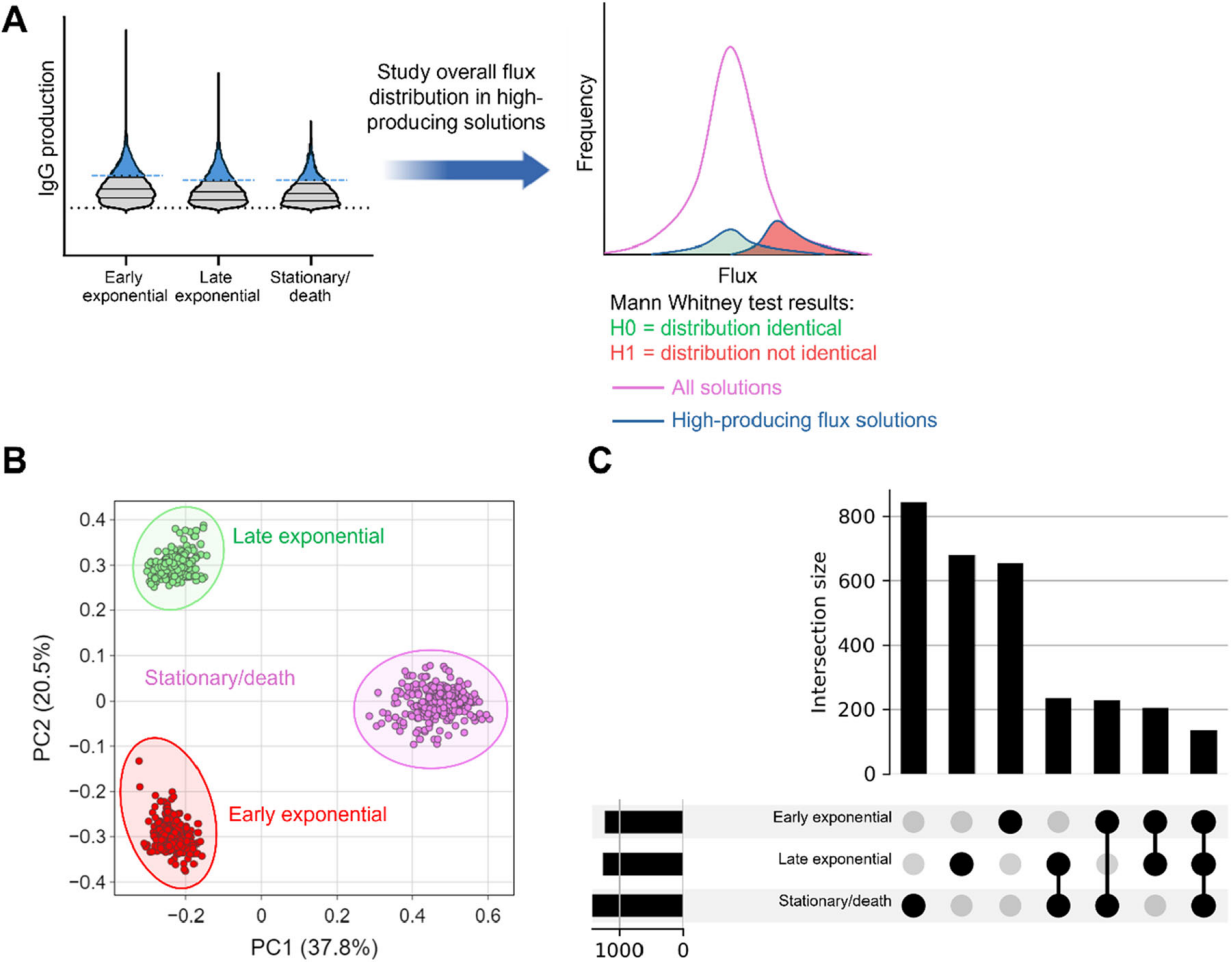
Isolating and studying high antibody‐producing flux sampling solutions. A. Workflow for isolating the “high mAb‐producing” solutions from flux sampling and determining whether the flux of individual reactions is skewed in these solutions, and therefore associated with high mAb production. B. PCA of the flux through total model reactions of the high‐producing solutions (top 95th percentile, as illustrated in A), for each time phase model. C. Upset plot to show the number of reactions associated with high mAb production for each culture phase model and where these overlap between separate culture phases. Associated reactions have been determined using a Mann–Whitney *U* test (FDR < 0.05).

The next step was to identify and isolate the highest mAb‐producing solutions, representing the 95th percentile. Metabolic flux predictions were compared from the 95th percentile to the general pool of 5000 solutions, using the Mann–Whitney *U* test (Figure 4A) to identify those reactions that change (with either increased or decreased flux) with increased mAb production. When the metabolic profiles (6336 reactions across 250 high mAb‐producing solutions) were visualised, it could be observed that the landscape of reaction fluxes was specific to the culture phase metabolic model, with distinct separation between the early exponential, late exponential and stationary/death phases (Figure 4B). Early and late exponential high mAb‐producing solutions show separation from the stationary/death phase along PC1 (37.8% of explained variance), while all the culture phases can be separated using PC2 (20.5% of explained variance) (Figure 4B).

Using results from the Mann–Whitney *U* test, it was possible to determine the number of reactions with a change in flux significantly associated with high mAb‐production, across the different culture phases and flux sampling solutions (Figure 4C). Results indicated that there were many reactions with a change in flux which was associated with high mAb production, some of which were unique to a specific culture phase, and others which were commonly associated across all three culture phases. There were the highest number of reactions uniquely associated with high mAb‐production at the stationary/death culture phase (841 reactions), suggesting either the upstream regulation or the downstream effect of high mAb production has the most unique profile later on in CHO cell culture (Figure 4C). In comparison, there were a similar number of reactions uniquely associated with high mAb production at the early and late exponential culture phases: 643 and 678 reactions, respectively (Figure 4C). There were 132 reactions associated with high mAb production that were common to all culture phases (Figure 4C). The high frequency of reactions predicted by models as being associated with high mAb production indicated that no single metabolic reaction or enzyme was driving improved production, and supported the high‐throughput, 'omics‐led approach of metabolic modeling.

**Figure 5:**
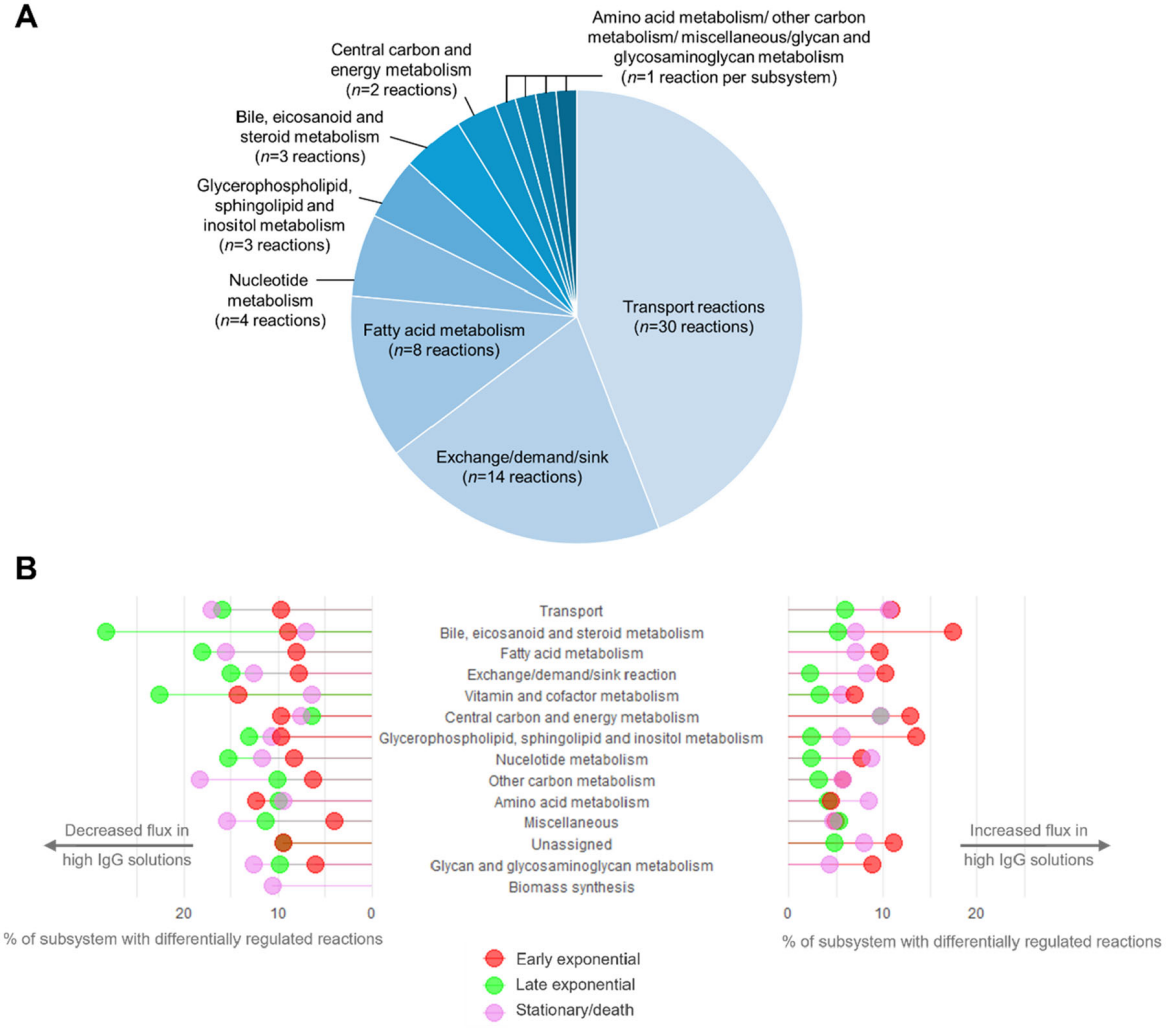
Metabolic subsystems associated with increased mAb production rates. A. The metabolic subsystem annotations of those reactions found to be associated with high mAb production, and which are shared across all culture phases (*n* = 132 reactions from Figure 4C) B. The proportion of total reactions in a subsystem which have had a change of flux associated with increased mAb production flux (decreased: left‐hand side, increased: right‐hand side). Data from flux sampling (*n* = 5000) solutions. Significance was determined using Mann–Whitney *U* test (FDR < 0.05). Colour of point refers to time phase model, as indicated in key. Notebook: Subsystem dot plot visualized on FluxSampling_highIgG_reactions_subsystemsVis.R.

Of those 132 reactions which were associated with high mAb production across all culture phases, there were 68 reactions with metabolic subsystem annotations in iCHO2441. Of these 68 reactions, there were 11 unique metabolic subsystems represented, with the most prominent being “Transport reactions,” “Exchange/demand/sink reactions,” “Fatty acid metabolism,” and “Nucleotide metabolism” (Figure 5A). Of those metabolite transport reactions which were associated with high mAb production, one‐third involved the transport of amino acids, therefore highlighting amino acid transport as a principal subsystem associated with mAb production. Other metabolites which were described by these “Transport reactions”; included fatty acids, molecules involved in redox signalling (namely thioredoxin and glutathione), nucleotides and hormones, such as l‐thyroxine.

Once the main metabolic subsystems had been identified, the direction of regulation of flux (i.e., upregulation or downregulation) associated with high mAb production was explored. Once the count of reactions per subsystem associated with high mAb production was normalised as a portion of the total metabolic subsystem, “Transport reactions,” “Bile, eicosanoid and steroid metabolism,” “Fatty acid metabolism,” and “Exchange/demand/sink reaction” were the top subsystems associated with high mAb production (Figure 5B). Overall, models predicted that the early and late exponential culture phases have contrasting metabolic flux profiles of high mAb production. Most subsystems had the highest proportion of upregulated reactions at the early exponential culture phase, while the late exponential culture phase models predicted that cells more often downregulate reactions to favor high mAb production (Figure 5B). Considering the regulation of specific subsystems, there was almost twice as high a proportion of reactions from the “Transport reaction” subsystem which were downregulated in the high mAb solutions for the late exponential and stationary/death culture phases as there were upregulated (Figure 5B). This indicated that the downregulation of some metabolite transport was associated with high mAb production at the later culture phases, where we would expect antibody to be being synthesised. The downregulation of specific metabolic reactions could explain an increase in mAb production by suggesting there is a re‐focus in the pathways through which energy is being transferred. Aside from transport reactions, models predicted the greatest proportion of reactions downregulated in high mAb‐producing solutions for the “Bile, eicosanoid, and steroid metabolism” subsystem, where roughly 30% of the entire subsystem showed downregulation with high mAb production at the late exponential culture phase (Figure 5B). Furthermore, between 15% and 20% of all fatty acid metabolism reactions were downregulated with high mAb production at the late exponential and stationary/death culture phases, while less than 10% of these reactions were downregulated at the early exponential culture phase, suggesting downregulation of fatty acid metabolism is associated with high mAb production more so at the later time points (Figure 5B). Other observations included the fact that “Vitamin and cofactor metabolism” showed variable levels of downregulation with high mAb production for each culture phase, and there was a roughly three times as great proportion of “Glycerophospholipid, sphingolipid, and inositol” reactions upregulated at the early exponential compared to both other culture phases (Figure 5B). Overall, the regulation of reactions associated with high mAb production showed differences across each culture phase, with specific culture phase‐specific signatures emerging.

### 3.4 Amino Acid Transport Changes Associated With High mAb Production Have Culture Phase‐Specific Signatures

**Figure 6:**
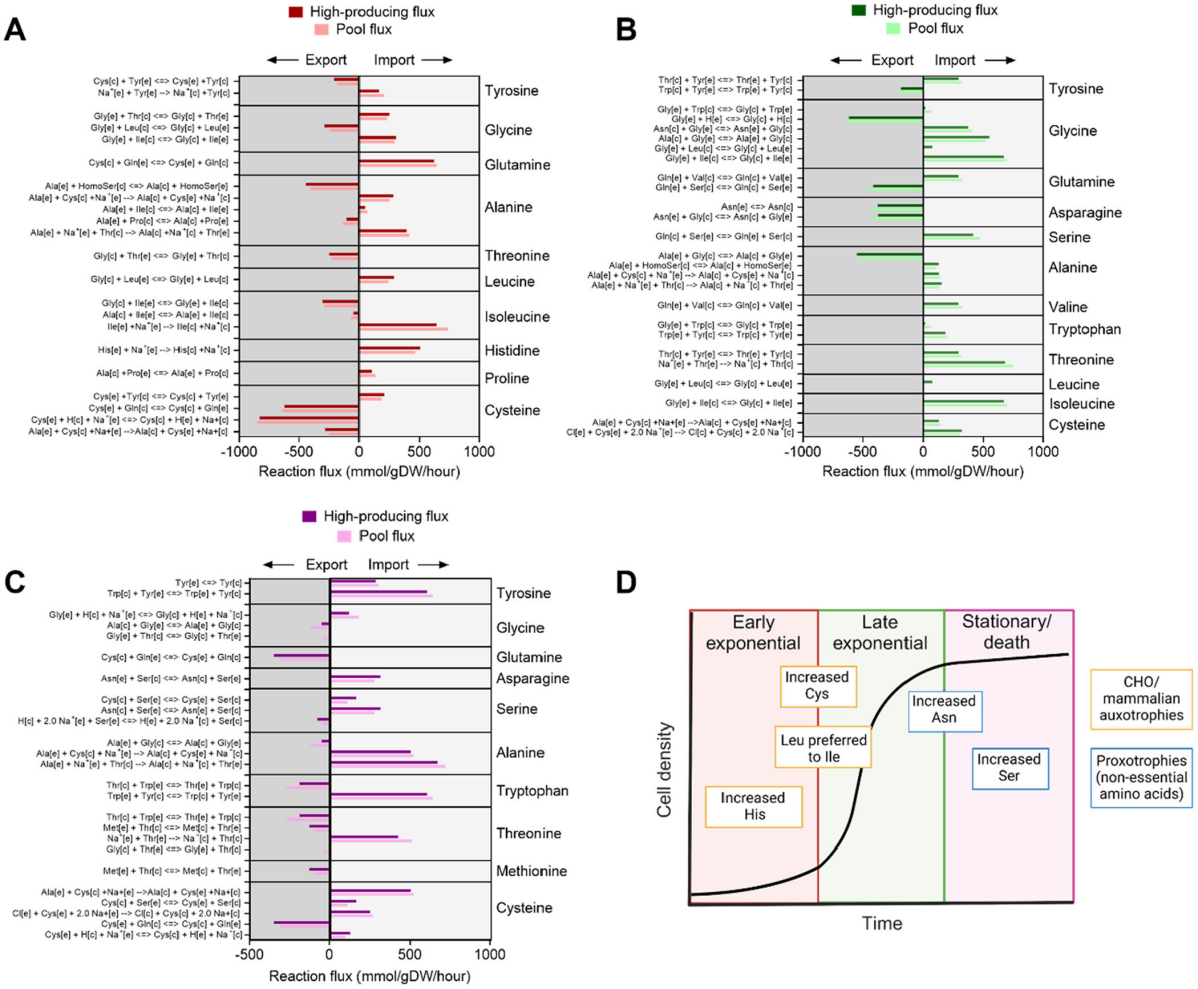
Proposed amino acid transport signatures for distinct CHO cell culture phases. A. Model‐predicted amino acid signature for the early exponential culture phase metabolic model. Amino acids have been grouped on the *x*‐axis; import has been shown in the right‐hand side of the graph as positive reaction flux (mmol/gDW/h); export has been shown in the left‐hand side of the graph as negative reaction flux (mmol/gDW/h; darker bars correspond to the mean reaction flux for the high‐producing 95th percentile of flux sampling solutions, while the paler bar corresponds to the mean reaction flux for the general pool of flux sampling solutions. Reaction formula have been shown in the left‐hand *x*‐axis, with amino acid three‐letter codes; “[c]”: cytosol, “[e]”: extracellular. For the amino acid of interest, the forward reaction in the left‐hand *x*‐axis has been written with forward flow from extracellular to cytosol. B. Model‐predicted amino acid signature for the late exponential culture phase metabolic model. C. Model‐predicted amino acid signature for the stationary/death culture phase metabolic model. D. Summary illustration of the model‐predicted amino acid uptake signatures which have been associated with increased mAb production using flux sampling. Amino acids have been overlaid onto a schematic for CHO cell growth in culture. Yellow‐lined boxes correspond to CHO or mammalian auxotrophies and blue‐lined boxes correspond to CHO proxotrophies.

The next step was to use model predictions to decipher a culture phase‐dependent amino acid transport signature with the aim of influencing medium optimisation strategies. There were over 600 reactions at the early and late exponential and over 800 reactions at the stationary/death culture phases which were altered in high mAb solutions (Figure 4C). The most dysregulated subsystem in the high mAb solutions was “Transport reactions” and as mentioned earlier, the type of molecule occupying most of these reactions related to amino acid transport (i.e., one‐third of transport reactions associated with high mAb production involved amino acids). Therefore, those reactions involving amino acid transport, and which were highly correlated with the principal component features (from Figure 4B), were used to propose culture phase‐specific amino acid transport signatures of high mAb production (Figure 6).

In total, the top 50 loadings for both PC1 and PC2 were compiled for this analysis. Of these 100 total loadings, there were 65 unique reactions which were significantly associated with high mAb production. There were 40 amino acid transport reactions contained within this subset, and these described 15 different amino acids across the three culture phase models. These 40 amino acid transport reactions were studied to develop amino acid transport signatures associated with high mAb production, and which showed a unique pattern of regulation across the culture phases (Figure 6). For the purpose of directing amino acid supplementation for CHO cell culture media, those amino acids which models predicted overall increased import in association with high mAb production will be focussed on here.

Using the principal component loadings and results from the Mann–Whitney *U* test, of the original 40‐reaction subset described previously, there were 22 amino acid transport reactions which were associated with high mAb production at the early exponential time phase (Figure 6A). There were 10 different amino acids described by this subset of transport reactions, with the highest frequency of reactions—four reactions—describing cysteine transport (Figure 6A). When the import and export predictions were considered together, models suggested that overall, the increased import of cysteine is favored at the early exponential phase by high mAb‐producing cells. This conclusion was reached because there was increased import of cysteine via the APC transporter (reaction ID: AAPAOC69) coupled with decreased export via the l‐cysteine/l‐glutamine reversible exchanger (reaction ID: CYSGLUexR) and cotransport with sodium and counter transport of a proton (reaction ID: CYSSNAT5tc) in the high mAb‐producing solutions (Figure 6A). In addition, the increased import of histidine was associated with high mAb production at the early exponential phase, involving sodium symport (reaction ID: HISt4) (Figure 6A). Another observation at the early exponential phase was that there could be overall less isoleucine transported into the cell, owing to decreased import (reaction ID: ILEt4) and increased export (reaction ID: AAPAOC11), coupled to the increased import of its structural isomer, leucine (reaction ID: AAPAOC6) (Figure 6A).

Moving onto the late exponential culture phase, in parallel to the early exponential model, results predicted that there was increased import of cysteine in high mAb‐producing solutions, through the CYSATB0tc reaction (Figure 6B). Another similarity between the early and late exponential culture phases was that there was decreased import of isoleucine into the cell (reaction ID: AAPAOC11) coupled with increased import of leucine (reaction ID: AAPAOC6) (Figure 6B). The late exponential phase model also suggested that the export of asparagine from the cell could be decreased in association with improved mAb production, exemplified by decreased flux in the high mAb‐producing solutions across two exporting reactions (reaction IDs: ASN_Lte and AAPAOC8) (Figure 6B). Decreased export of asparagine from the cell favors increased intracellular concentrations of this amino acid in high mAb‐producing cells.

Finally, the stationary/death phase was studied for amino acid signatures associated with high mAb production (Figure 6C). On top of the decreased export of asparagine at the late exponential phase, this behavior was observed at the stationary/death phase, where there was increased import of asparagine associated with high mAb production (reaction ID: AAPAOC42) (Figure 6C). Another amino acid which was associated with high mAb production at the stationary/death culture phase, but which was unique to this culture phase, was serine, where there was increased import via two separate reactions (reaction IDs: AAPAOC39 and AAPAOC42) (Figure 6C).

In summary, “Transport” reactions was defined as a key subsystem associated with high mAb production following enrichment analysis and modeling has suggested multiple reactions describing six different amino acids: cysteine, histidine, leucine, isoleucine, asparagine, and serine which may show a culture phase‐dependent signature and could be explored by media optimization experiments (Figure 6D). Of these six amino acids, four are essential to CHO DG44 cells and need to be included in the feed (cysteine, leucine, isoleucine, and histidine) and asparagine and serine are non‐essential amino acids, which CHO DG44 cells can produce themselves (Figure 6D).

## 4 Discussion

This study adapted the iCHO2441 (Strain et al. 2023) GEM to FDBKA, an industrially relevant mAb producing CHO cell line cultured in a fed‐batch bioreactor. The goal was to predict metabolic signatures and amino acid uptake patterns, associated with distinct culture phases. Constraints were applied to the iCHO2441 GEM based on transcriptomic data, and flux sampling was used to compare different culture phases and identify high mAb‐producing subpopulations. Our analysis revealed multiple metabolic subsystems linked to improved mAb production, with a particular emphasis on metabolite transport and amino acid metabolism. Six specific amino acids (His, Cys, Leu, Ile, Asn, and Ser) were identified as key targets for future optimization.

To validate these predictions and further optimize mAb production, we propose targeted experiments that perturb media conditions during specific culture phases. By tailoring nutrient availability to the metabolic needs of the cell cultutre at different stages of the bioreactor process, we aim to further enhance mAb productivity.

### 4.1 Transcriptomics Analysis and Bioprocess Validation Support Reliable Constraint‐Based Models

Initially, transcriptomic analysis identified three distinct culture phases based on gene expression patterns (Figure 2), aligning with the recognised early exponential, late exponential, and stationary/death phases of CHO cell culture. Previous studies have linked transcriptomic signatures to these culture phases, with late exponential phase characterised by differential regulation of DNA synthesis, protein transport, and the cell cycle leading to an increase in cell proliferation and cell size increase (Pan et al. 2017, 2019). Our results corroborate these findings, showing differential regulation of DNA replication, RNA transport, and the cell cycle pathways between the early and late exponential time points (Figure 2). This congruence between transcriptomic data and GEM constraints indicates the identification of relevant and expected pathways through functional enrichment.

Regarding the validation of flux sampling predictions against bioprocess data (Figure 3), a strong agreement was observed for the majority of sampling predictions and experimental bioreactor measurements. This provides confidence in the suitability of these models for intracellular flux analysis. Notably, the models accurately captured the decrease in growth rate over time and the consistent mAb production during the bioreactor process. However, the model failed to predict lactate consumption during the late exponential phase, potentially due to the transitional nature of this phase. This suggests that averaging expression data across multiple days for GEM model constraint may limit the predictive capability for metabolic reactions that exhibit significant metabolic shifts. This limitation is not unique to our study, as similar challenges have been observed in other bioprocess modeling studies, particularly in capturing the transition from lactate production to consumption during late exponential phase. For example, a systematic evaluation of 'omics integration algorithms showed that models tended to predict a higher (more positive) rate of lactate production than was measured experimentally (Machado and Herrgård 2014).

### 4.2 The Stationary/Death Phase of CHO Fed‐Batch Culture Has a Unique Metabolic Signature

The high mAb‐producing flux sampling solutions demonstrated a culture phase dependent metabolic phenotype (Figure 4). Notably, the stationary/death phase was represented by more than 800 unique reactions, associated with high mAb production, significantly more than the early and late exponential phases (~600). This is expected, given the bioprocess evidence showing that the uptake and secretion behavior of the FDBKA cells changes the most dramatically between the early exponential and stationary/death phases.

Enrichment analysis of flux sampling solutions identified metabolite transport (where 30% of these reactions described amino acid transport), fatty acid metabolism, nucleotide metabolism, and lipid metabolism as the subsystems most strongly associated with increased mAb production (Figure 5). While the changing dynamics of glucose and amino acid consumption have been extensively studied in CHO protein production (Coulet et al. 2022), nucleotide metabolism has received less attention. However, the accumulation of glycerol‐3‐phosphate and glycerol (contained in the glycerophospholipid, sphingolipid, and inositol subsystem in iCHO2441) in the stationary/death has been previously reported (Coulet et al. 2022). Additionally, when normalised for subsystem size, our results highlighted central carbon and energy metabolism as a top‐ranked subsystem associated with increased mAb production. This aligns with previous studies demonstrating cell engineering strategies targeting genes involved in the tricarboxylic acid cycle can enhance mAb productivity (Zhang et al. 2021). Furthermore, overexpression of the mitochondrial pyruvate carrier and pyruvate oxaloacetate has been shown to increase mAb production (Zhang et al. 2021).

### 4.3 Amino Acid Consumption Behavior Regulates mAb Production and Varies With CHO Fed‐Batch Culture Phase

Media optimisation is a well‐established approach to maximise protein production in CHO cell culture. A previous study applied FVA on the iCHO1766 CHO GEM and predicted that threonine and arachidonate would be effective media supplementations. This prediction was validated in vitro resulting in a a two‐fold increase in mAb production (Fouladiha et al. 2020). Our work further supports this approach by predicting a preference for leucine uptake over isoleucine in high mAb‐producing solutions (Figure 6). This aligns with the observation that balancing leucine and arginine is crucial for controlling mAb productivity due to their presence in the protein product (Fan et al. 2015). Consequently, the six amino acids highlighted by our modeling analysis, along with 14 others, are included in the stoichiometric equation for the mAb product expressed in this study. Due to this direct relation between amino acid demand of a CHO cell line and its specific product profile, it is important to note that the findings made in this study are specific to the FDBKA CHO cell line and product formulation and should be investigated using a parallel workflow before being translated to other cell line scenarios.

Research has indicated that amino acid consumption, which impacts mAb productivity, can be influenced by the expression of amino acid transporters. For instance, the upregulation of genes encoding transporters of alanine, cysteine, and glutamate during the stationary phase, has been linked to increased glutathione synthesis, a process associated with high mAb production (Kyriakopoulos et al. 2013). This upregulation is thought to be a response to decreasing intracellular concentrations of amino acids (Kyriakopoulos et al. 2013).

Further evidence for a tailored feed strategy comes from studies demonstrating changes in amino acid consumption behavior over time. Non‐essential amino acids like asparagine, glutamine, and cysteine exhibit high consumption during the exponential growth phases (Pan et al. 2017). Additionally, the consumption of essential amino acids is more closely tied to cell number than cell volume (Pan et al. 2017). These findings support the notion of a phase‐specific feed strategy, as certain amino acids, such as arginine, can accumulate towards the later culture phases, indicating an oversupply in the fed‐batch medium (Coulet et al. 2022).

However, it is essential to consider the solubility limits and potential toxic or inhibitory effects of amino acid additions. Excessive supplementation at any given time point should be avoided, as exemplified by the association between asparagine supplementation and ammonia accumulation (Pereira et al. 2018). Previous studies also advise that levels of leucine and serine above 0.5–1 mM could result in growth inhibition (Pereira et al. 2018) and high cysteine concentrations can lead to oxidative stress and reduced bioprocess performance (Komuczki et al. 2022). However, negative impacts of excessive isoleucine and histidine have not been reported.

## 5 Conclusions

This study has provided valuable insight into the dynamic metabolic changes that occur during CHO cell fed‐batch bioreactor culture, however, several questions still remain. A critical future direction is to elucidate the causal relationships between these metabolic shifts and improved mAb production. Specifically, it is essential to determine whether alterations in amino acid consumption are a direct driver of increased mAb synthesis or a secondary consequence of cellular adaptation in a bioprocess environment.

To address this, carefully designed experiments involving tailored media formulations and genetic engineering approaches are necessary. By manipulating specific amino acid uptake and metabolic pathways, we can directly assess their impact in mAb productivity. Constraint‐based modeling will provide a powerful tool to predict and simulate the effects of these genetic and metabolic perturbations, and although these modeling predictions can only be theoretical before experimental mechanisms and evidence have been provided, this study highlights the hypothesis‐generating potential of constraint‐based models while prioritising the experimental validation of initial predictions to bioprocess data.

It is important to acknowledge that cellular responses to media composition can be complex and dynamic, involving feedback mechanisms and adaptation strategies that are not fully captured by current models. Therefore, a synergistic approach combining computational modeling and experimental validation will be essential to fully unravel the underlying metabolic mechanisms.

In conclusion, the findings presented in this study highlight the potential of metabolic engineering and systems biology to optimise mAb production processes. By leveraging advanced modeling techniques and experimental validation, we can develop more precise and effective strategies to enhance biopharmaceutical manufacturing.

## Author Contributions

K.E.M. and J.W. developed the methodology, analysed data, and wrote the manuscript. S.R., E.H., A.P., T.M., and L.P. generated data and assisted in the interpretation of results. M.R., A.J.D., and J.M.S. edited the manuscript and supervised the project. All the authors have read and approved the final manuscript.

## Supporting information

Figure 1. PCA of time series transcriptomics including Day 5.
