# Supplementary material for: Flux Sampling Suggests Metabolic Signatures of High Antibody‐Producing CHO Cells
Source: Biotechnol Bioeng. 2025 Apr 11;122(7):1898–913. doi: 10.1002/bit.28982 (PMC12152534; doi:10.1002/bit.28982)
Supplement: Supplementary file 1 — Figure 1. PCA of time series transcriptomics including Day 5. [file BIT-122-1898-s001.docx]

Supplementary figures

Time-series transcriptomics measurements were taken at each day between Day 4 and 14 of a fed-batch culture. Upon visualisation using PCA, there was a suspected batch effect at Day 5, therefore this sample was removed from further analysis. Day 5 was separated from all other days along PC2 (22.49%).


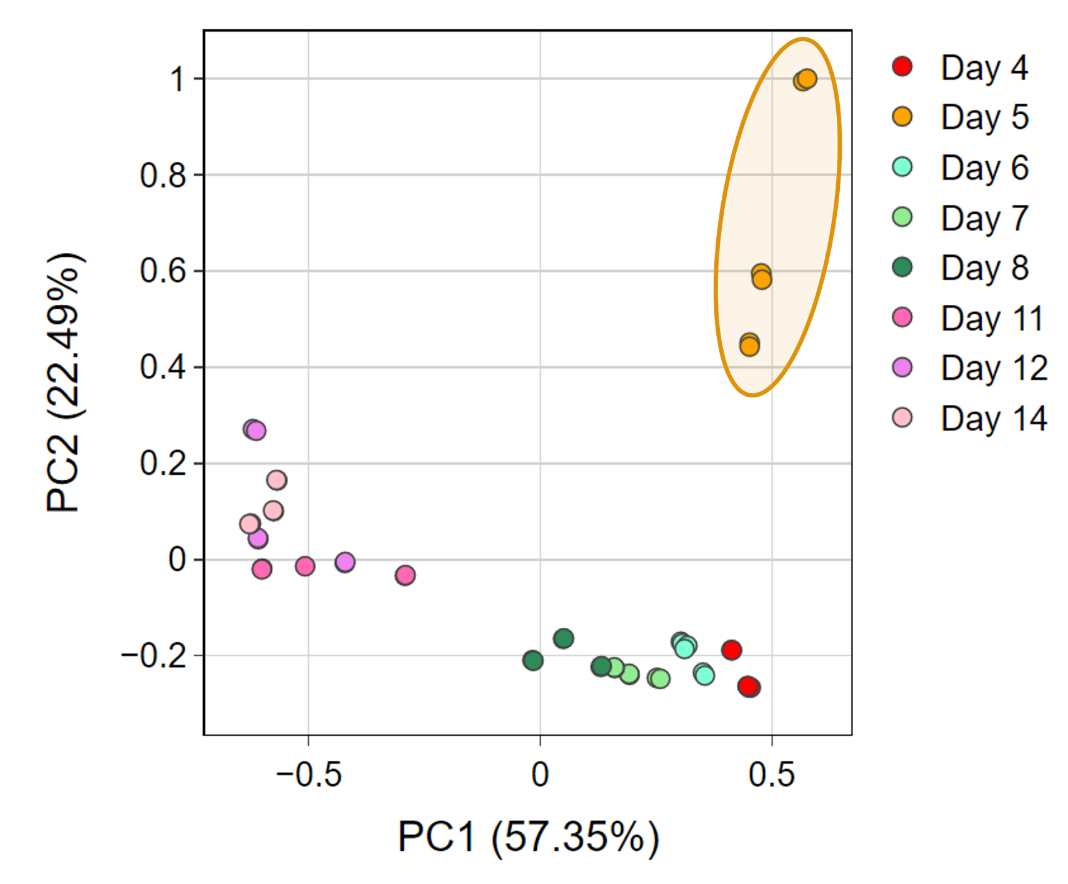


**Figure 1. PCA of time series transcriptomics including Day 5.**


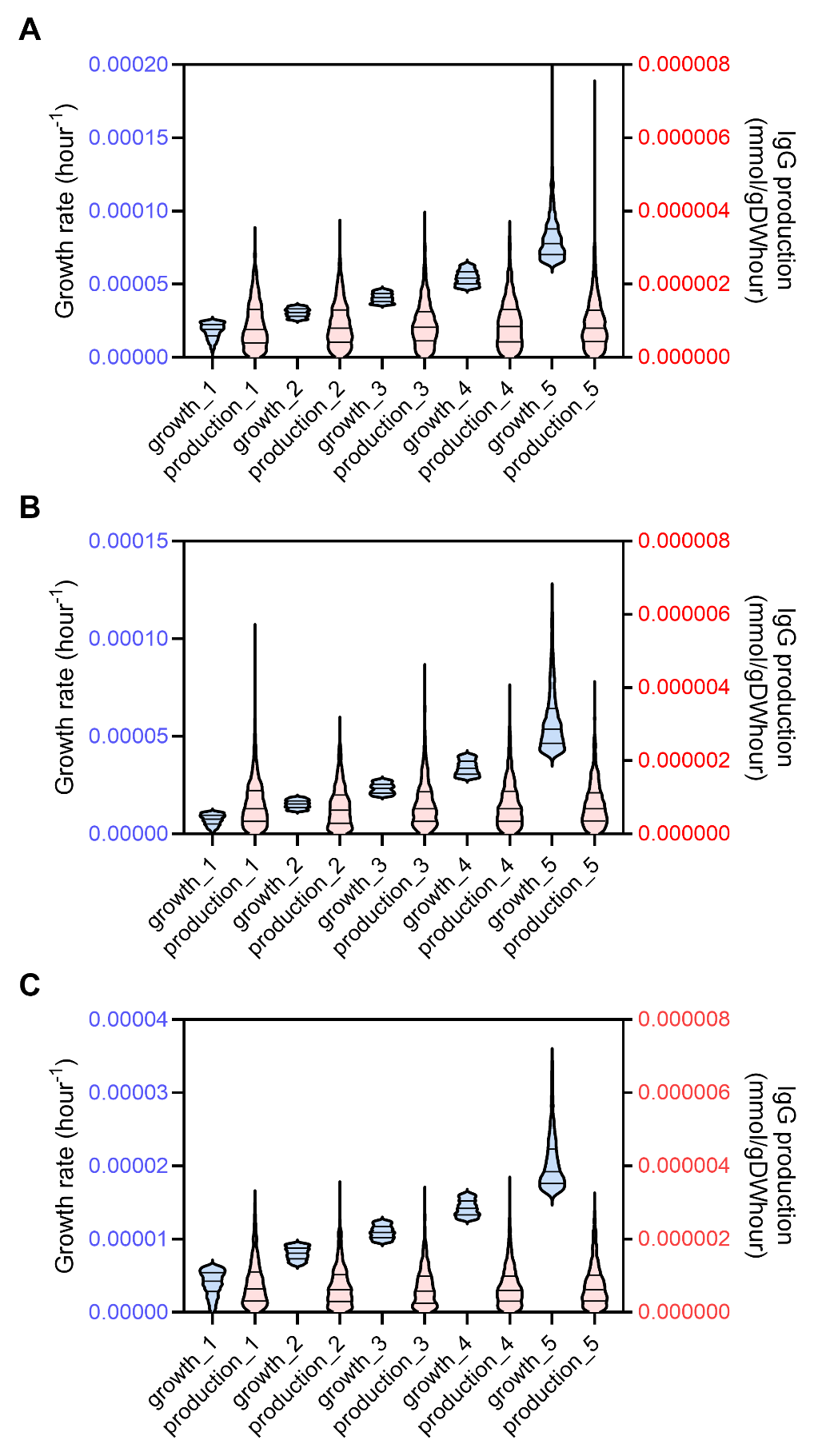


**Figure 2. Growth and IgG production do not correlate in the FDBKA cell line.**

Violin plots to show the relationship between growth rate (‘biomass_cho_prod’) and IgG production (‘ICproduct_Final_demand’) in flux sampling. Growth rate solutions (blue) have been organised into bins according to value, and the corresponding IgG production solutions (red) have been plotted alongside (i.e. ‘growth_1’ and ‘production_1’ originate from the same 1,000 flux sampling solutions. **A.** Early exponential culture phase model **B.** Late exponential culture phase model **C.** Stationary/death culture phase model.
